# Food cleaning in gorillas: Social learning is a possibility but not a necessity

**DOI:** 10.1371/journal.pone.0188866

**Published:** 2017-12-04

**Authors:** Damien Neadle, Matthias Allritz, Claudio Tennie

**Affiliations:** 1 School of Psychology, University of Birmingham, Birmingham, United Kingdom; 2 School of Psychology and Neuroscience, University of St Andrews, St Andrews, United Kingdom; 3 Department of Early Prehistory and Quaternary Ecology, University of Tübingen, Tübingen, Germany; University of York, UNITED KINGDOM

## Abstract

Food cleaning is widespread in the animal kingdom, and a recent report confirmed that (amongst other behaviours) wild western lowland gorillas also show food cleaning. The authors of this report conclude that this behaviour, based on its distribution patterns, constitutes a potential candidate for culture. While different conceptualisations of culture exist, some more and some less reliant on *behavioural form* copying, all of them assign a special role to social learning processes in explaining potentially cultural behaviours. Here we report the results of an experiment that tested to what extent food cleaning behaviour in a group of captive Western lowland gorillas (*Gorilla gorilla gorilla*) relies on social learning processes. Subjects were provided with clean and dirty apples. When they were provided with dirty apples, all subjects showed evidence of food cleaning in at least 75% of trials. Preferred cleaning techniques differed between individuals, four out of five of subjects expressed a behaviour analogous to that reported in wild conspecifics. Given this occurrence of food cleaning in a culturally unconnected population of gorillas, we conclude that social learning is unlikely to play a central role in the emergence of the food cleaning behavioural form in Western lowland gorillas; instead, placing a greater emphasis on individual learning of food cleaning’s behavioural form.

## Introduction

Cleaning food is common in the animal kingdom. There is evidence of the general behaviour of removing contaminants from food (using water or not) in many animal species, including: wild boars [1], Japanese macaques [2–4], vervet monkeys [5] crab eating macaques and capuchin monkeys (both by [6]), along with all species of great ape [7]. The removal of unpleasant contaminants from food that is about to be consumed is likely adaptive—the repeated consumption of dirt, sand or grit (or indeed any hard contaminate) can wear down and/or damage the teeth [8], in addition to not providing any or much benefit when ingested. Finally, removing contaminants from food may aid in avoiding parasite infection [4].

In a recent contribution exploring the wild behavioural repertoire of Mountain (*Gorilla gorilla beringei*) and Western (*Gorilla gorilla gorilla*) gorillas, Robbins et al [9] identify food cleaning as “customary” (see below) in several wild populations of Western lowland gorillas. Robbins et al. apply the so-called method of exclusion across five gorilla (*Gorilla gorilla sp*.) field sites: three western lowland gorilla sites (Moukabala-Doudou National Park, Gabon; Bai Hokou, Central African Republic & Mondika, Republic of Congo) and two mountain gorilla sites (Karisoke Research Center, Rwanda & Bwindi Impenetrable National Park, Uganda). Their research combined observations of the gorillas at these research sites between 1998 and 2015 (19 groups of western lowland gorillas and 12 groups of mountain gorillas). These extensive, collective observations of gorilla behaviours were used to generate a list of 41 behaviours (through discussion by researchers) that may differ across groups. The occurrence of these behaviours was then assessed across sites following the methods of previous studies that also applied the method of exclusion to great apes [10,11]. Behaviours were classified as ([9] pg. 5):

1. “Customary: occurring in most or all members of at least one age-sex class.
2. Habitual: observed in several individuals, but does not meet criteria for customary.
3. Present: observed, but neither customary or habitual.
4. Absent: Not observed, but due to any apparent ecological reason
5. Ecological explanation: Behaviour is absent because of a lack of a key ecological feature i.e. absence of a required plant in the environment.”

As a result of this cross-site comparison, Robbins et al [9] identified 23 “potentially cultural traits” from the total 41 observed behaviours: 12 within species variants (with variation between sites of one species of gorilla) and 11 between species variants (with variation *only* observed between mountain and western lowland gorillas). Also documented are three “universal” traits, 10 “rare behaviours” and five traits whose absence in one species could be explained by ecological variations.

Amongst the list of “potential cultural behaviours” only one food processing behaviour was noted, namely fruit cleaning. This behaviour will be the focus of this article. Robbins et al ([9] pg. 8) describe fruit cleaning as: “Rubbing fruit against [the] arm or body, presumably to remove dirt; for some fruit it may be to remove spines” [9]. Based on this definition it can be assumed that the “form” of the behaviour [12] is the rubbing of fruit on the body to remove an unwanted substances/parts. It is possible that removing spines from fruit and removing dirt from fruit constitute two separate behaviours. However, action required is to rub the food against the arm or body, the *form* of the behaviours would be identical—merely the target *substance* to be removed would be different. Thus, here and below we will refer to the behavioural form of rubbing food against the body or extremities as *food cleaning*.

Food cleaning was observed at all western lowland gorilla research sites; however, it was never observed at mountain gorilla research sites. This pattern led the authors to conclude that this behaviour is potentially cultural on the basis of “variation between mountain and western gorillas” ([9] pg. 8).

The concepts of culture and social learning are closely related. A factor common to many (if not all) definitions of “culture” is the presence of social learning, thus, it becomes possible to equate the two terms, i.e., for a behaviour to be considered “cultural” it must be influenced by social learning—which will be the definition of culture we will use here (similar to [13]). However, social learning is a broad term that encompasses many different mechanisms [14] and to consider all mechanisms as equal may gloss over important distinctions between types of culture (see below). In the literature relevant to our discussion, the term culture is also often understood by outcomes. And so, the term culture is also often employed by those who apply the method of exclusion and refers to “group typical behavioral patterns shared by community members” with the inference “that {they are} to some degree {…} reliant on socially learned and transmitted information” ([9] pg. 8). Note that Robbins et al [9] define additional criteria in order for a behaviour to be considered cultural: for them (and all applications of the method of exclusion) the behaviour must be customary and present in one population, whilst being absent in another (first mentioned in [10]).

Alternatively, it is possible to use a definition that further divides the concept of culture, such as that put forward by Tomasello [15]. Tomasello and others distinguished between cumulative culture (often argued to be uniquely human) on the one hand, and non-cumulative culture on the other. Non-cumulative culture, in this framework, can still emerge and benefit from social learning but lacks certain *specific* forms of social learning (usually the claim is for a lack of imitation (see [15] but also see [16] for a contrasting view; see [17] & [18] for some evidence that other, special, forms of social learning may also suffice for cumulative culture). Importantly, over time cumulative cultural processes lead to behaviours beyond that which any individual within the species could innovate within their own lifetime [19]; for example, a space shuttle or a, more basic, motor car. In this paper, we will use the, more general, definition of culture (equating it with social learning) as no doubt remains that social learning is present in non-human animals.

A common method inferring influences of social learning (and thus, inferring culture) is the *method of exclusion* (also known as the ethnographic method, see [9–11]). This method regularly serves as a first point of reference for researchers who want to explore whether a behaviour is *cultural* (as defined above). It is a useful method because it produces a list of behaviours, the prevalence and distribution of which can most parsimoniously be explained by an influence of *social learning*, rather than being due purely to ecological and/or genetic factors. The method of exclusion thus aims to exclude pure genetic and/or environmental level differences.

Practitioners of the method of exclusion have attempted to control for ecological confounds by identifying when behaviours are ecologically not possible, i.e., when a required artefact or situation necessary for a given behaviour is not present (for example, fruit availability being permanently limited in a particular ecology would be an ecological explanation for fruit cleaning being absent, [9]). Applications of the method of exclusion typically use ecological explanations as a simple dichotomous category, i.e., the behaviour is ecologically possible or not possible (e.g. [9–11]).

Besides ecology, genetic differences may also play a confounding role when applying the method of exclusion. Generally, the most convincing cases, to date, that claim for cultural (rather than environmentally and/or genetically induced) differences between great ape field sites are in cases where populations of the same subspecies live in close proximity to each other—i.e. in very similar environments–*and* with the possibility of a high likelihood of genetic mixing, i.e. lacking physical barriers [20]. The fact that differences can and do still exist in these cases (e.g. [20,21]) makes the conclusion that social learning influences these differences very likely. These are the currently best supported cases for culture in (wild) great apes.

However, even in these remaining, and most convincing cases, there is always the possibility that subtle differences at the genetic and/or environmental level are ultimately responsible for the observed differences. Thus, even the most fine-grained method of exclusion (e.g. [20]) may, at be best, regarded as a method that uncovers which behaviours *may* be cultural (which should be verified through captive studies). However, this method cannot in itself detect what/which type(s) of social learning is/are at work, in particular whether social learning is necessary to explain the *form* of the target behaviour. Determining the underlying mechanisms thus requires controlled experimentation, which is most often attained in captive settings.

In this study, we test for the presence of food cleaning in a group of captive western lowland gorillas. We chose food cleaning because it is the only foraging related behaviour for which a claim of cultural transmission was made, in the most recent application of the method of exclusion to the case of gorillas [9] (foraging behaviours are amongst those more easily simulated in captivity). Such a claim makes it necessary to test in captivity whether the behavioural form of food cleaning is in fact more likely the product of individual rather than social learning (thus “cultural” in the strong sense); this is achieved by testing a captive group that has never had any contact with any of the wild groups for whom the behaviour has been reported (by [9]), or have not been trained by humans to show the target behaviour. Should the behaviour be observed, and in different forms, within such a captive group, it would appear more plausible that individual learning is sufficient to explain the form, and perhaps even frequent re-innovation of the target behaviour. Note that given the likely high prevalence of somewhat dirty food it is implausible to think to discover totally dirty-food naïve subjects. Instead, here we test for evidence of a *culturally unconnected population-level* instance of food cleaning to add to the cases reported by Robbins et al [9]. It is possible, though unconfirmed, that at least two of the three wild populations reported by Robbins et al [9] were culturally unconnected (given the substantial geographic distance (approx. 890km) between them (Moukalaba-Doudou national park vs. Mondika, Dzanga-Sangha national park); thus, this sample constitutes a, potential, third population observed for food cleaning. Here, culturally unconnected refers to the fact that the individuals have not had contact with a member of a population previously shown to perform the target behaviour. For example, our sample of western lowland gorillas have not been in contact with any of Robbins et al’s [9] samples, thus, social learning between the populations is not possible.

In this study, we provided captive Western lowland gorillas with affordances that may encourage food cleaning (i.e., by provisioning them with peeled apples that were covered in sand). We recorded instances of food cleaning behaviours and categorised the specific methods of cleaning. The results of these experiments are discussed in the light of the data presented by Robbins et al. [9].

## Method

The recordings used in this study are taken from a larger set containing data for all genera of great apes; here we only re-coded the gorilla recordings (all subjects were western lowland gorillas, [6]). Forty-eight recordings of gorillas being exposed to dirty (sand covered) peeled apples (1 hour 50 minutes = 24 recordings) and clean peeled apples (1 hour 1 minute = 24 recordings; total across both apple types = 2 hours 51 minutes) were re-coded for this study; this was done with the goal to code for food cleaning (described by [8]), a behaviour which was not analysed in the previous study [7]. The data contained no evidence of food washing, i.e. cleaning by using water (as coded for by [7]) in any trial despite there always being an open basin of fresh water available. The present study used the video recordings from Allritz et al [7]; however, all data reported in this article was obtained by applying a novel coding system designed specifically for investigating the behaviour of food cleaning. This means that no data from the original study was used during analysis or to form conclusions, making this study independent of the previous one.

### Subjects

The subjects in the selection of videos were five Western lowland gorillas (1M/4F; mean age at time of testing: M age = 16.60, SD = 12.58; both years) located and housed together at the time at the Wolfgang Köhler Primate Research Centre in Leipzig Zoo, Germany (see Table 1 for details of apes along with familial relations; these gorillas comprised the entire group of gorillas at WKPRC at the time data was collected). One subject, Zola, a three-year-old female juvenile was also tested, however her data was excluded from further analyses. This was because Zola was always tested together with her mother, who monopolised virtually all food.

**Table 1:** Subject rearing information, italicised mother/father indicates that parents were *not* involved in this study.

Subject	Sex	Year of birth	Place of birth	Mother/Father
Bebe	Female	~1979	Wild, Cameroon	*Unknown*
Gorgo	Male	1981	Krefeld, Germany	*Boma/Massa*
Viringika	Female	1995	Zurich, Switzerland	*Inge/N’Gola*
Kibara	Female	2004	Leipzig, Germany	Virginika/Gorgo
Louna	Female	2006	Leipzig, Germany	Bebe/Gorgo
Zola	Female	2008	Leipzig, Germany	Virginika/Gorgo

No subject was deprived of food or water and all participated in the research voluntarily. All individuals in the sample were captive born, apart from one female, “Bebe”, who was wild born. “Bebe” was captured in Cameroon (rather than from the Republic of Congo or Gabon, as the populations in Robbins et al’s [9] study). Given that there is no reported claim, to our knowledge, for wild gorillas in Cameroon displaying food cleaning it is unlikely that the subject “Bebe” socially learned this behaviour from a wild conspecific. Thus, it is likely that, even if we did not capture the very first reinnovation of this behaviour (here reinnovations are defined as described in [22]), any noted (re)innovation would be the result of individual learning as it could not have been transmitted, via social learning, from a wild population.

In accordance with the recommendations of the Weatherall report “The use of non-human primates in research” all the gorillas were housed in semi-natural indoor and outdoor enclosures containing climbing structures, such as ropes and platforms; and natural features, such as vegetation, trees and streams. They received regular feedings, primarily consisting of vegetables, had access to enrichment devices including shaking boxes and poking bins, and water ad lib. Subjects voluntarily participated in the study and were never food or water deprived. Research was conducted in the observation rooms.

No medical, toxicological or neurobiological research of any kind is conducted at the Wolfgang Koehler Primate Research Center. Research was non-invasive and strictly adhered to the legal requirements of Germany. The study was ethically approved by an internal committee at the Max Planck Institute for Evolutionary Anthropology (members of the committee are Prof. M. Tomasello, Dr. J. Call, Dr. D. Hanus, veterinarian Dr. A. Bernhard, head keeper F. Schellhardt and assistant head keeper M. Lohse). Animal husbandry and research comply with the “European Associations of Zoos and Aquaria Minimum Standards for the Accommodation and Care of Animals in Zoos and Aquaria”, the “World Association of Zoos and Aquariums Ethical Guidelines for the Conduct of Research on Animals by Zoos and Aquariums” and the “Guidelines for the Treatment of Animals in Behavioral Research and Teaching” of the Association for the Study of Animal Behavior. IRB approval was not necessary because no special permission for the use of animals in purely behavioural or observational studies is required in Germany.

### Experimental conditions

All trials were conducted between 08:00 and 12:30 (by MA), and subjects completed one trial per testing day. Subjects were tested in a separate testing room (2.52 x 2.61m). They were video recorded from the time that they entered the room; recording ceased once all three apples had been consumed or after 10 minutes. Each individual was tested four times in each condition. In both conditions, there were three peeled apples placed in the centre of the testing room; subjects were deemed to be motivated to consume the apples, based on experimenter experience of the food preferences of these individuals. The variation between conditions was the state of the apples, in the dirty condition the peeled apples were rolled in play sand (Redsun branded; a fine-grained quartz sand) designed for children and deemed free of harmful substances by TUV Nord, Germany, in testing. Further, the sand was dried in an oven for approx. 20 minutes at 200°C before the apples were covered in it. Three gorillas of the final sample began with four sessions of the clean apples condition followed by the dirty apples condition, and two gorillas began with the dirty apples condition before completing the clean apples condition.

### Data coding

DN coded all recordings using the categories in Table 2. To assess the interrater reliability of the coding, a randomly selected 25% of these sequences were further analysed by another independent coder (HH). This random sample was stratified to ensure that reliability was attained for at least one video for each gorilla in each condition. The chosen videos were randomly selected by assigning numbers 1–4 for each trial in each condition and then randomly generating numbers between one and four to coincide with a video for reliability analysis. Cohen’s Kappa Coefficient [23] was computed based on agreement between both coders. The results are reported below. Reliability was assessed based on whether the coders agreed on the method of cleaning employed (as described in Table 2). Instances of food cleaning were coded into bouts for ease of reliability analysis. A new cleaning bout was coded if the individual:

1. set the apple on the floor for more than 5 seconds
2. swapped method of cleaning
3. swapped hand used for cleaning
4. moved from one apple to another (because of eating the apple or abandoning it)
5. Ceased cleaning for more than 30 seconds
6. Took a bite from the apple

**Table 2:** Methods of cleaning fruit coded, along with description, as provided to independent coders, see S1 File.

Method	Description
Palm	Rubbing the apple with, on or between the palm(s) of the hands
Back of hand	Rubbing the apple with the back of the hand (this does not include rubbing ON the back of the hand)
Forearm	Rubbing the apple on the hair of the forearm or the of back of the hand, this is distinct from back of hand in that the apple is being moved in this case whereas, in the former, the hand is moving.
Finger	Rubbing the apple with one finger.
Substrate	Rubbing the apple on any substrate (e.g. the floor)

## Results

### Frequency of food cleaning

**Fig 1:**
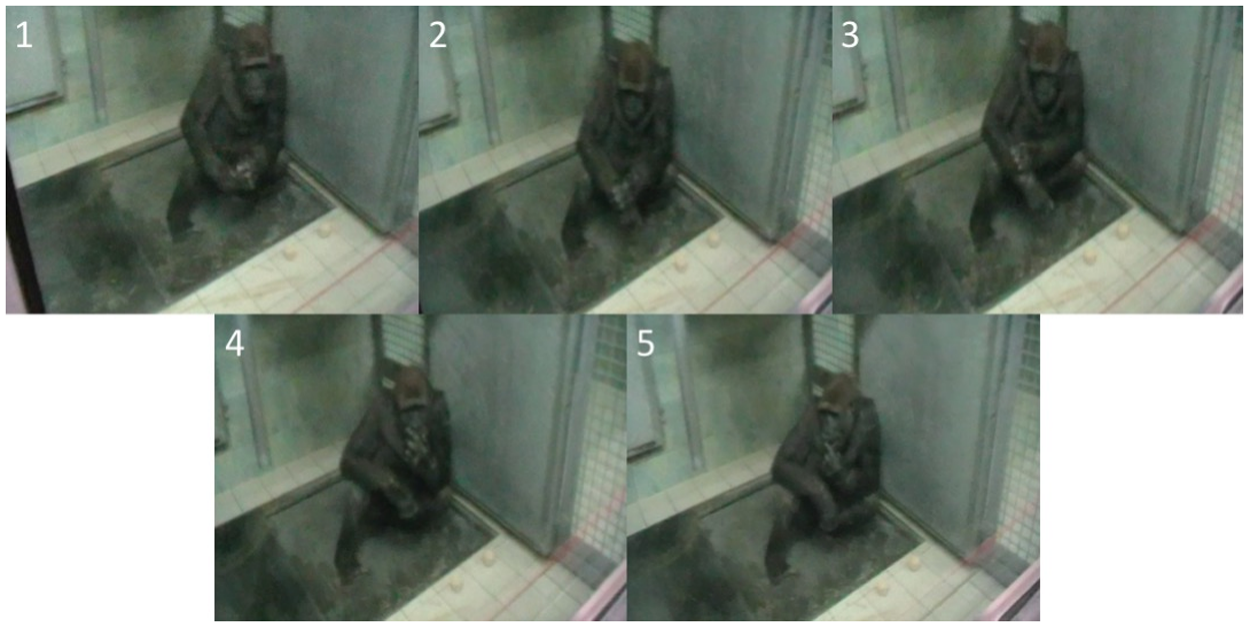
Food cleaning sequence in an adult female Western lowland gorilla (“Kibara”) cleaning and eating a peeled sand covered apple using the method described by Robbins et al [9]; See S2 File.

The results of a Cohen’s Kappa Coefficient test show that both coders achieved a satisfactory level of agreement (κ = 0.71, SE = 0.07, *p* <.001) in the *method* (i.e., which technique) of cleaning used (see Table 2); an example of a typical food cleaning sequence is shown in Fig 1. Both coders agreed fully in their classifications of forearm (the target behaviour based on [8]) and also that no cleaning behaviour occurred in any of the clean apple trials.

**Fig 2:**
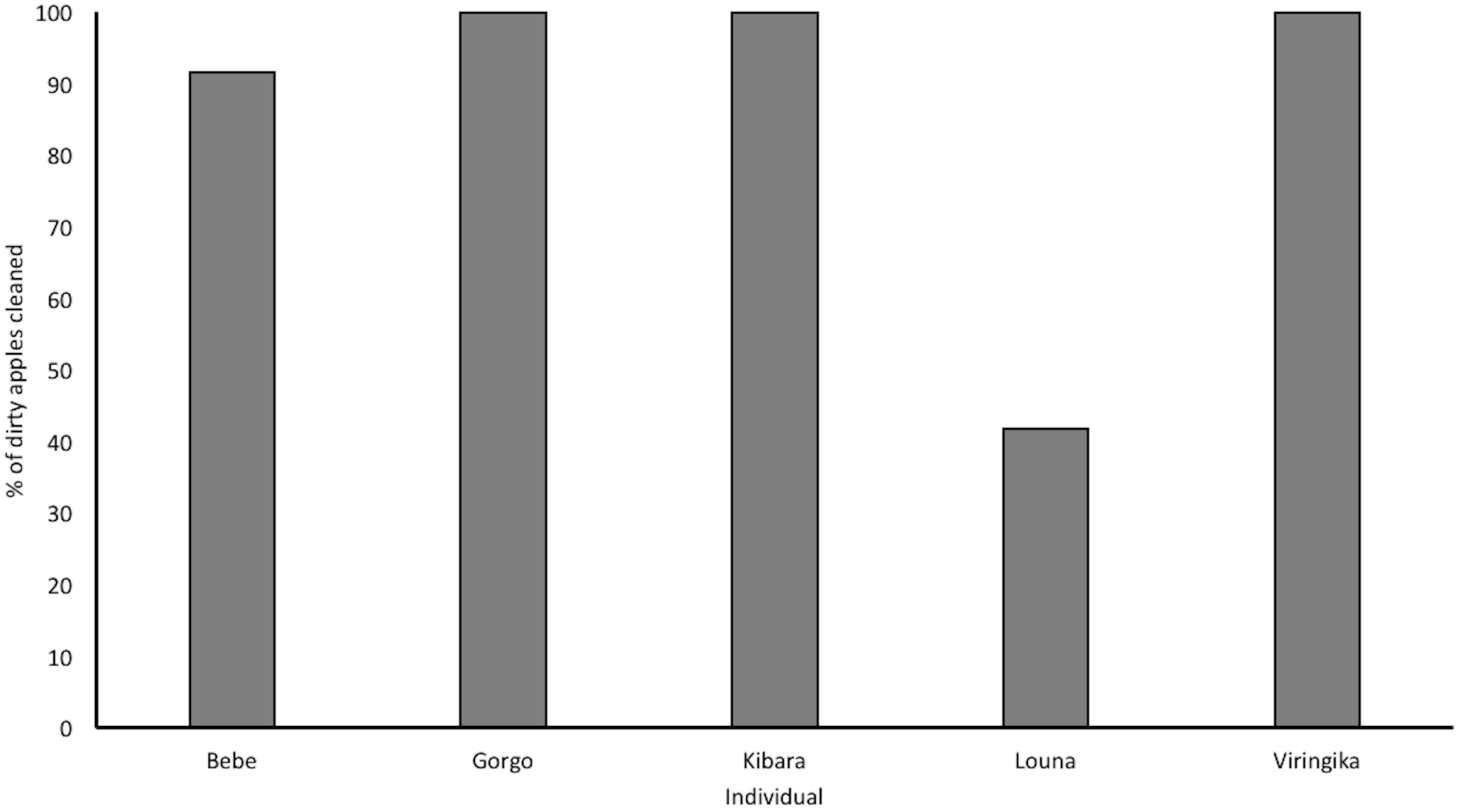
Percentage of dirty apples cleaned *before* first bite was taken, separated by individual.

The results showed that this sample of Western lowland gorillas expressed food cleaning behaviour in 95% of dirty apple trials and in 0% of clean apple trials. Only in a single trial were all the apples eaten whilst still dirty, this trial was the first exposure of Louna (3-year-old female) to the dirty apple condition. All gorillas expressed food cleaning at least once in at least 75% dirty apple trials. Not all subjects attempted to clean all dirty apples; from a total of 60 dirty apples, 8 were eaten uncleaned (apples were eaten uncleaned by Bebe (1 apple in 1 trial; 8.3% of total dirty apples) and Louna (7 apples in 4 trials; 58.3% of total dirty apples)), see Fig 2.

**Fig 3:**
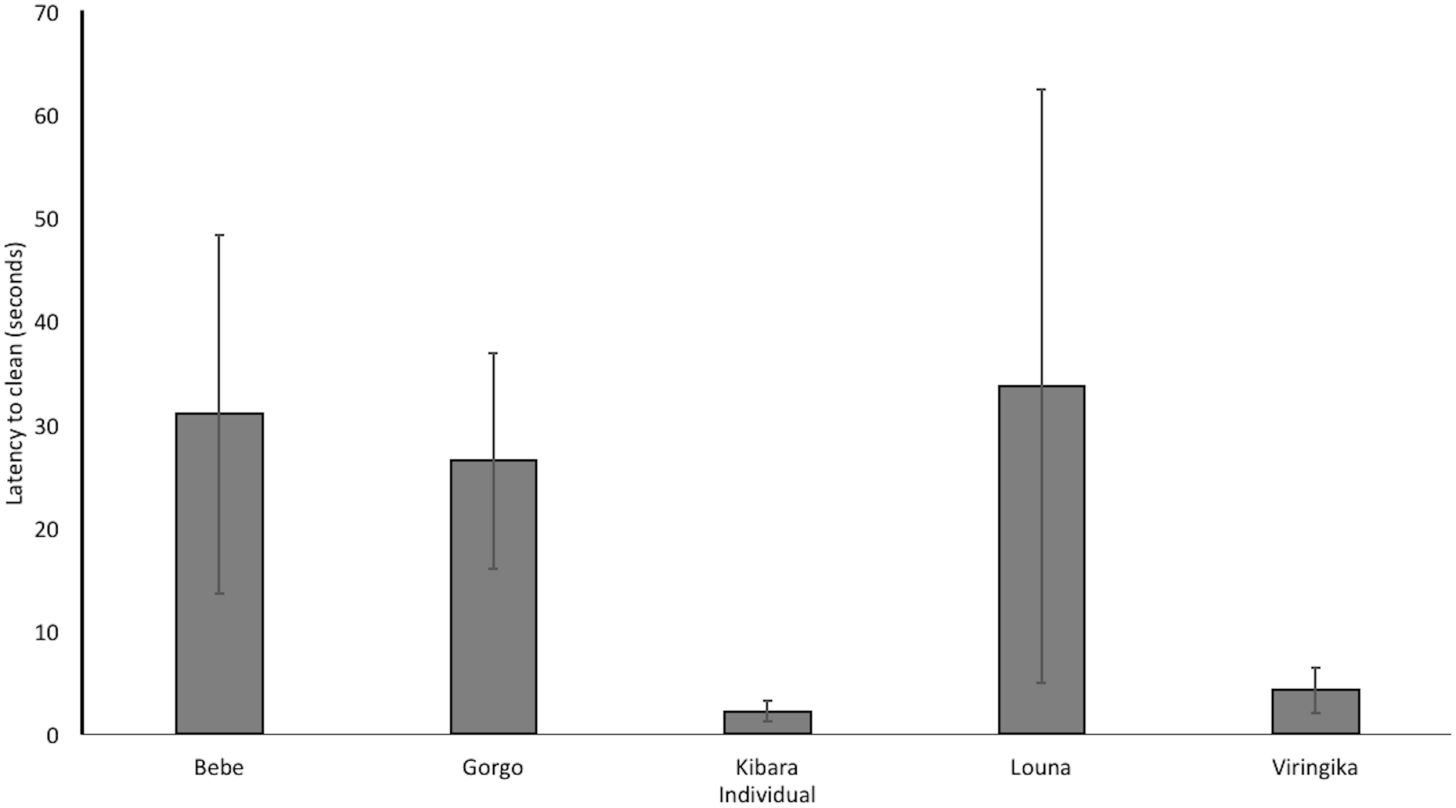
Mean individual latency to begin cleaning. Showing variation between subjects, error bars indicate ± 1 standard error of the mean.

The mean latency (time passed since entering the test room) to first clean an apple was 19 seconds (SD = 27 seconds). Average individual latency to clean apples differed substantially between subjects, ranging from one second to 91 seconds (across trials; see Fig 3).

### Methods of food cleaning

**Fig 4:**
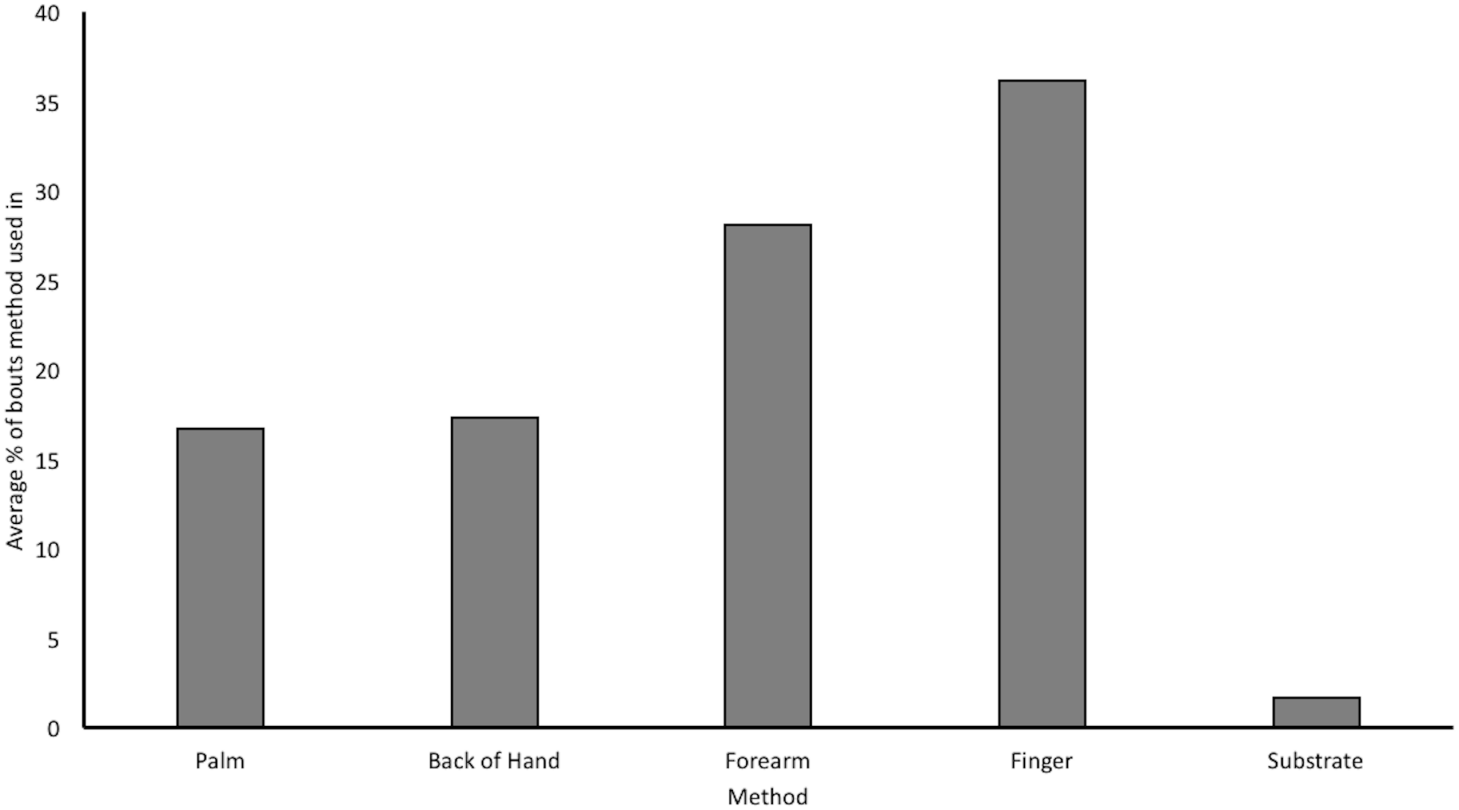
Bars indicate average % of times that a method was used to clean the apples, data implicates all individuals across all trials.

**Fig 5:**
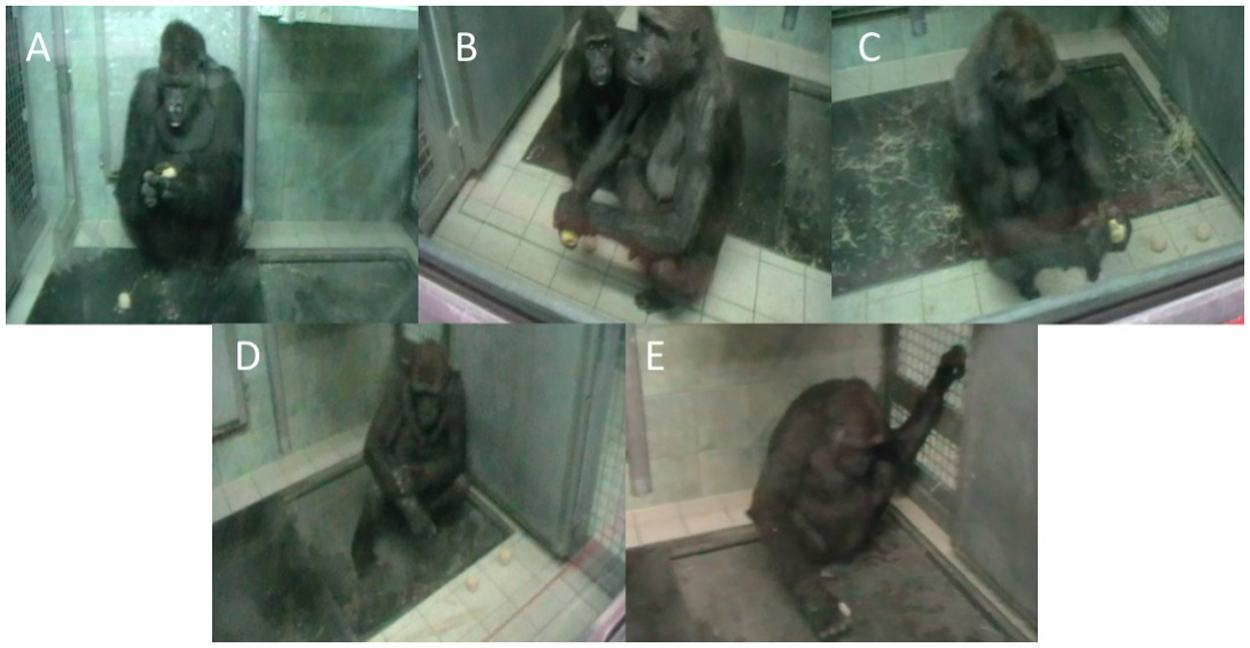
Five methods of food cleaning used by Western lowland gorillas in this study: A) Finger B) Palm C) Back of Hand D) Forearm E) Substrate; see S1 File for video recordings.

The methods of cleaning the fruits used in this data, listed in order of frequency of bouts, were *finger* (41%), *back of hand* (20%) *palm* (20%), *forearm* (18%) and finally *substrate* (1%); see Fig 4. For examples of the cleaning methods used, see Fig 5 and supplementary material (S1 File). The more frequent expression of the *finger* technique is owing to the way that bouts were coded, meaning that if an individual swapped hand, a new bout began. This meant that the *finger* cleaning method (which frequently swapped from using the left index finger to the right) often resulted in a high number of bouts (see raw data; S3 File). The *palm* and *back of hand* methods were coded more frequently for a similar reason; Viringika often used these techniques in quick succession, e.g. slapping the sand off the apple with the palm followed by the back of the hand and vice versa. As a result, this inflated the values. The frequency data serves to highlight individual differences, see Table 3.

**Table 3:** Number of bouts using each method, divided by individual.

	Bebe	Gorgo	Kibara	Louna	Viringika	Total
Palm	2	0	3	1	**38**	44
Back of Hand	5	0	3	0	37	45
Forearm	**8**	0	**22**	2	8	40
Finger	1	**82**	4	**4**	1	92
Substrate	0	0	3	0	0	3
Total	16	82	35	7	84	224

*Note*. Figures in bold show the most commonly used method by each individual.

As can be seen from Table 3, there were substantial differences in individual technique preference.

## Discussion

We report here a behaviour markedly similar, in underlying form, to that reported by Robbins et al [9], which we here coded as forearm technique. Our results show that food cleaning was present in an unconnected sample of captive western lowland gorillas. We also show a degree of individual variation in methods used to clean contaminants from dirty food, with a further four methods being expressed.

The data presented here shows another populational instance of food cleaning occurring in western lowland gorillas, specifically in a new, that is, culturally unconnected, captive population. Given the fact that access to some dirty food can be assumed to be a common feature of wild and captive populations it is implausible to expect to test a truly dirty-food naïve subject. Instead we argue that being culturally unconnected to any populations in which the behaviour is reported is sufficient to assume that the studied species—if it shows the behaviour under such circumstances—would have had to reinnovate target behaviour from scratch—i.e. without the help of cultural transmission (logically this would then have happened in *at least* a single naïve individual of the tested group). This said, we acknowledge here that it is possible that this study did not capture the original first reinnovation(s) of food cleaning (this could have happened prior to the study, given the ubiquitous access to dirty food). However, given the underlying logic just outlined, i.e., that at least one individual had to thus reinnovate it in our population at some point, this timing issue is inconsequential to the argument we are making here (i.e. of whether it is *possible* for a reinnovation of the behaviour to occur without social learning as a crutch). Our findings thus show that food cleaning can be reinnovated without cultural help by naïve western lowland gorillas. This finding indicates that the form of the behaviour is not heavily reliant on social learning (as is often practically inferred by readers of the term “culture”). Instead these findings are consistent with individual learning (including genetic predispositions) being most important in the emergence of this behaviour, a fact that is consistent with food cleaning being a “latent solution” for Western lowland gorillas, derived through a process of individual learning [24,25]; however, it is likley that social learning facilitates the harmonsiation of the initial reinnovation of the behaviour within a population.

This conclusion is drawn based both on the data presented here, and the data presented by Robbins et al [9], which shows that all western lowland gorilla groups that these authors observed for food cleaning have been documented to show it. Indeed, the captive group in the present study displayed a behaviour identical to that described by Robbins et al [9]; which led us to assume that the *behavioural form* is most parsimoniously explained by individual learning rather than by cultural transmission. Although low fidelity social learning mechanisms (such as stimulus enhancement) may be implicated in catalysing the harmonisation of food cleaning within populations (i.e., increasing overall *frequencies*), social learning of any type does not appear to be necessary for the emergence of the *form* across individuals.

Robbins et al [9] applied the method of exclusion to identify potentially cultural behaviors, however, the interpretation of their results is complicated by differences between the groups of gorillas for whom they collected data, both with regard to genetic variation [26] and with regard to ecology [27]. It is possible that the failure of mountain gorillas to express food cleaning may be a result of a combinations of genetic variation and ecological differences. It is possible (although unlikely) that the genetic differences between the two subspecies of gorillas [26,28] lead to distinct behavioural phenotypes (though see below for alternative views). This would not at all need to imply a “direct genetic code” for food cleaning, rather that the cognitive abilities that lead to cleaning foods (and likely also other behaviours) might have become advantageous at some point and thus were selected for. This may have happened in conjunction with the frequency increasing effects of low fidelity social learning mechanisms (i.e. mechanisms that can enable “behavioural drive” effects (see [29,30]).

However, given the abundance of food cleaning in primates [2–7], a perhaps more plausible argument is that the differences in mountain and western lowland gorilla *ecology* are the root (or main) cause of this difference. It is possible that wild mountain gorillas do not show food cleaning because their relevant ecology differs from that of western lowland gorillas [27]. If this were true then, if wild mountain gorillas *were* to share the same ecology as western lowland gorillas, and if they were thus forced to consume more food that would benefit from cleaning; then they may also independently innovate food cleaning. Indeed, the feeding ecology of mountain gorillas and western lowland gorillas seems indeed to differ quantitatively in relevant ways, namely the relative presence of dirty fruit (an item that is associated with food cleaning, both in this study and in a wild sample; [9]). The mountain gorilla data presented by Robbins *et al* [9], is taken from Karisoke Research Centre, Virunga Volcanoes Rwanda and Bwindi Impenetrable National Park, Uganda. At Karisoke there are no large fruiting trees within the mountain gorillas range [9] and fruit accounts for <1% of the total time spent foraging [31] and only 11% at Bwindi [32]. In contrast, up to 70% of Western lowland gorilla foraging time centred around fruit [33] and 98% of faecal samples contained evidence of fruit consumption [34]. And so, there would seem to be much more opportunity for lowland gorillas to encounter dirty fruit, especially as mountain gorillas, being in effect primarily folivores [31], are more likely to consume growing plants [35]. Latent solutions have previously been implicated in the processing of growing plants (e.g. nettles) in gorillas [25]. These plants may be less likely to be encountered in a dirty state but even if this is not true, they would likely be too arduous to clean individually, i.e. leaf by leaf (as would be implied by the food cleaning method). And so, mountain gorillas may very well be able to reinnovate food cleaning; but their current ecology may neither provide sufficient opportunities [36], nor a necessity [37] for them to do so. There are no captive mountain gorillas, and so an experimental study like ours, to address this question, is not possible.

Thus far, there is no evidence that food cleaning in western lowland gorillas requires social learning to occur at all. Yet, whilst our target behaviour does not require social learning, it is possible that behaviours that only very few individuals can innovate (therefore must pass via social learning in order to become local traditions) do exist. All candidate cases (and here the method of exclusion is indeed a very good starting point) require testing in ideally fully culturally unconnected populations, to determine whether these cases do *require* social learning. Therefore, testing captive, unconnected individuals under controlled conditions and exposing them to the material used in (and for) the target behaviour (but without demonstrations of behavioural form) can make an important contribution to our understanding of how these behaviours may or may not spread within wild communities.

It is established that some primates have cultures (i.e., that they can be influenced by social learning of some sort(s)), however culture—defined in this way—is nothing special to primates; instead it is common in the animal kingdom. Such minimal cultural forms range from insects [38] to birds [39] and reptiles [40], to cetaceans [41] and rodents [42]–to name a few. The question whether non-human animals have culture that resembles human, i.e., cumulative, culture [43] remains open. Some scholars believe this type of culture to be reserved to humans [15]. It is possible that this seeming uniqueness may be related to the need for high fidelity social learning in order for exact behavioural forms to be maintained (and improved on in cumulative culture) successfully [19,24,44,45]. This said, researchers disagree on which mechanisms are at the heart of this process, the classical claim is that imitation is required for culture to accumulate [43,46], whilst more recent experimental evidence suggests that end-state emulation (the copying of environmental end-results of behaviour) may sometimes be sufficient, at least (again) in the human case [17,18].

No matter the exact underlying mechanism, cumulative culture *requires* higher fidelity social learning in order to be maintained (though the mechanism is unclear; [24,44]), this is clearly not the case in food cleaning (as it seems that for its form to appear, no social learning at all is *required*, see above). This is worth noticing, as potential cases of culture in animals are also potential cases of cumulative culture.

Yet, while food cleaning has proven not to be a case of cumulative culture, we turn next to the question of whether it fulfils the less demanding criterion of culture. Rather than rely on a definition of culture that assumes that social transmission of form (i.e. social learning) is a *necessary* factor, we here use a new definition, as we have shown evidence to the contrary. This—often implicit—assumption that social transmission is essential in all forms of culture comes from the working assumption that individual learning (and thus, reinnovations) is/are not at the heart of many of the behaviours observed in wild primate populations. Given mounting evidence that this alternative is a very real possibility [12,22,24,25] we believe that a more general, i.e., conservative definition is more suitable. Thus, resulting in a “soft” test for *any* culture based around a, new, very general definition: a behaviour can be considered cultural, if social learning (of any form, including learning from artefacts) plays any role at all in the form *and/or* the frequency of the behaviour (and/or any produced artefacts, compare also “tradition”; see [47]). This is similar to other definitions in use, e.g. to Kendal, Kendal, Hoppitt and Laland, who define culture (in its broadest form) as “any instance of social *transmission* of behaviour regardless of the underlying social learning processes” ([13] pg. 1; emphasis added). Note however, that their definition implies a transmission of the behavioural form. We hesitate to restrict cultural status to those behaviours (and species that express those behaviours) that *require* social learning to *transmit* a behavioural form. This is because there may be a large difference between these two cases, which benefits from explicit naming, cases that do constitute evidence of behavioural form transmission should be explicitly named as such (once evidence of transmission is found, until such a time our soft definition of culture should suffice). Our more basic definition does not require the transmission of behavioural forms: mere increases in frequency of the behaviour suffice (thus also allowing the appearance of the behaviour to be merely catalysed across individuals through social learning—and which might be commonly the case in non-human animals). Given the plethora of claims for culture in various species (with no evidence of behavioural form copying [38–41]) we believe this, soft, definition better reflects the current state of secure knowledge of non-human animal culture.

The currently available data on food cleaning behaviour in gorillas (as described here and in 9) do not show a clear signature of any social learning. And so, the behavioural form, *as well* perhaps as its distribution and frequency, could also be explained through individual learning. Still, we also lack the data that would show a lack of an influence of social learning on reinnovating likelihood across individuals. And so, low fidelity social learning may still serve as a catalyst for increases in frequency of this behaviour within populations. Thus, whilst we have shown that social learning is not required for the expression of food cleaning in gorillas (resulting in cultural status defined as by [13] being rendered void) when we apply our new definition of culture, the possibility remains that food cleaning represents a cultural trait (in this minimal, soft, sense).

Whiten et al ([48] pg. 1494) defined “behaviours recorded as absent at no sites studied” as “species universals”. Given that food cleaning has been shown to occur in each sample of western lowland gorillas that have been tested/observed for it to date ([8] and this study), it fulfils this definition of a species universal, a notion incompatible with that of culture, according to Whiten et al [48]. However, even species universals *can* be socially learned ([49] pg. 84). Thus, and again depending on the definition of culture applied, it is possible, using our “soft” definition, to ascribe a cultural status even to these species universal behaviours.

In sum, we have demonstrated that individual learning, in reacting to ecological settings, likely plays a key role in the emergence of food cleaning behaviors across different groups of gorillas. Moreover, we showed that the individual form of the behaviour (and other, related, behaviours) can vary considerably between subjects within the same group. We acknowledge that social learning (in some form, most likely low-fidelity) may still play a role in facilitating the spread of food cleaning behaviors within some groups—thus potentially fulfilling a “soft” definition of culture, as we presented above. Overall, we believe that the current data, considered in conjunction with that presented here, does not show the signature of behavioural form transmission (though other low fidelity forms of social learning may be present); without this transmission, it is impossible to consider this behaviour similar to human culture. Therefore, given the ability of western lowland gorillas to express food cleaning (primarily) through individual reinnovation, we consider that food cleaning is better classified as a latent solution sensu Tennie et al [24].

## Supporting information

S1 File All methods of food cleaning reported in this study. (MP4)

S2 File Forearm method of food cleaning, similar to that described by Robbins et al (2016). (MP4)

S3 File Raw data. (XLSX)
